# Advances in the Treatment of Ulcerative Colitis—From Conventional Therapies to Targeted Biologics and Small Molecules

**DOI:** 10.3390/ijms27031534

**Published:** 2026-02-04

**Authors:** Aleksandra Wilk, Mateusz Pawłowski, Ewa Balcerczak, Agnieszka Jeleń, Marek Mirowski, Dagmara Szmajda-Krygier

**Affiliations:** 1 Department of Pharmaceutical Biochemistry and Molecular Diagnostics, Medical University of Lodz, Muszynskiego 1, 90-151 Lodz, Poland; aleksandra.wilk@student.umed.lodz.pl (A.W.); mateusz.pawlowski@stud.umed.lodz.pl (M.P.); ewa.balcerczak@umed.lodz.pl (E.B.); agnieszka.jelen@umed.lodz.pl (A.J.); 2 BRaIn Laboratories, Medical University of Lodz, Czechoslowacka 4, 92-216 Lodz, Poland

**Keywords:** ulcerative colitis, inflammatory bowel disease, novel biologic drugs, small molecules

## Abstract

The goals of ulcerative colitis (UC) treatment are focused on improving quality of life, achieving steroid-free remission, and minimizing the risk of cancer. In UC traditional management, a step-up strategy involves introducing increasingly more immunosuppressive medications, thus avoiding unnecessary overexposure to more potent drugs. However, in cases of severe, acute UC, priority is rapid and effective treatment to minimize the risk of complications such as bleeding, intestinal perforation, toxic megacolon or the need for colectomy. Modern approach to UC management shifts to an “accelerated step-up” or “top-down” approach in high-risk patients to prevent bowel damage. A holistic approach—integrating molecular research, clinical management and patient-centered care—enhances our understanding of disease mechanisms and therapeutic strategies, ultimately supporting improved outcomes and overall quality of life. This review aims to present the treatment options for UC along with an overview of the most modern therapies and experimental agents.

## 1. Introduction

Ulcerative colitis (UC) is a form of inflammatory bowel disease (IBD) marked by recurrent episodes of inflammation to the mucosal layer of the colon, typically beginning in the rectum and potentially spreading proximally throughout the large intestine [1]. The illness results in ulceration of the colonic lining with a characteristically sharp demarcation between inflamed and healthy mucosa [2]. Although UC inflammation primarily affects the mucosa, it can also involve deeper layers of the bowel wall. In chronic, severe cases, the colon may become shortened and rigid, losing its normal haustral pattern, which results in the appearance of a ‘lead pipe’ visible on imaging. Additional changes include proliferation of perirectal fat, widening of the presacral space and alterations in the vasa recta, which can persist even during remission. Microscopically, advanced UC is marked by thickening of the lamina propria and submucosa due to fibrosis, hypertrophy of the muscularis mucosa, fat deposition, thinning and relaxation of the muscularis externa [3].

The etiology of all IBDs is multifactorial, involving both genetic susceptibility and environmental triggers. In recent years, growing evidence has confirmed the heritable nature of inflammatory bowel disease [4]. Genome-wide association studies (GWAS) have identified over 200 genetic loci linked to IBD with 41 loci specific to Crohn’s disease, 30 unique to UC and 137 shared by both disorders [5]. Several genetic, immunological, microbial and environmental factors contribute specifically to UC. First-degree relatives of UC patients are approximately four times more likely to develop the disease with a positive family history. UC affects men and women equally and typically has a bimodal age distribution, with peaks between 15 and 29 years and between 50 and 80 years [6]. Key biological factors in UC include disruption of the intestinal epithelial barrier, immune system dysregulation and imbalance of the gut microbiota (dysbiosis). Environmental and lifestyle factors, such as mode of delivery, breastfeeding, antibiotic use, air pollution, diet, lack of physical activity, stress and tobacco smoking habits also influence disease risk. Additional risk factors include urban living, recent infections with *Salmonella*, *Clostridioides difficile* or *Campylobacter*, tobacco cessation and high consumption of sugary products. Conversely, protective factors comprise appendectomy, active smoking and having been breastfed during infancy. Excessive antibiotic use can disrupt gut flora, promoting dysbiosis and increasing vulnerability to inflammation, while smoking paradoxically appears to reduce UC risk and offers some protection, although it must be mentioned that smoking carries major prohibitive systemic health risks, despite the fact that nicotine may present anti-inflammatory effects [6,7]. Ulcerative colitis symptoms include urgency, incontinence, fatigue, frequent stools, mucus and abdominal cramps with less abdominal pain than in Crohn’s disease. Severe cases may involve fever and weight loss. Classification depends on the extent of colon involvement [8,9]. Clinical remission is defined as the resolution of key symptoms such as rectal bleeding and increased stool frequency and it is considered a key therapeutic target due to its association with mucosal healing. Endoscopic remission, characterized by minimal or absent mucosal inflammation, is a critical treatment target in UC, associated with better long-term outcomes, including reduced relapse, hospitalization and cancer risk. It is commonly assessed using scoring tools like the Mayo Endoscopic Subscore (MES) or Ulcerative Colitis Endoscopic Index of Severity (UCEIS) with complete mucosal healing (MES 0) offering the best long-term outcomes [10]. Among non-invasive biomarkers, fecal calprotectin (FC) is particularly useful in UC due to its superior correlation with endoscopic inflammation compared to C-reactive protein (CRP) or erythrocyte sedimentation rate (ESR). FC is also superior to CRP, since the latter can be normal up to 25% of patients with active disease. Although FC levels can be influenced by various factors unrelated to disease activity, it remains a reliable marker for predicting relapse and guiding treatment decisions when interpreted in clinical context [10,11]. Fecal biomarkers such as calprotectin and lactoferrin play a growing role in clinical practice, aiding in distinguishing IBD from functional disorders, assessing disease activity, monitoring treatment response and anticipating flares for timely intervention [12]. Disease activity in UC is commonly assessed using the Mayo scoring system, which supports treatment monitoring and decision-making. The primary therapeutic goals include achieving steroid-free remission, improving patients’ quality of life and reducing long-term complications such as colorectal cancer [13]. A holistic approach to UC—integrating molecular research, clinical management and patient-centered care—enhances our understanding of disease mechanisms and therapeutic strategies, ultimately supporting improved outcomes and overall quality of life [14]. This article aims to present the treatment options for UC along with an overview of the most modern therapies and experimental agents.

## 2. Conventional Treatment Options

### 2.1. Mild to Moderate Activity—5-Aminosalicylic Acid (5-ASA)

5-aminosalicylic acid (5-ASA) compounds represent the cornerstone of therapy for mild to moderate UC. Due to their well-established anti-inflammatory properties and favorable safety profile, 5-ASA formulations are widely regarded as first-line treatment options at inducing and maintaining remission in affected patients [8]. Studies indicate that most patients—between 88% and 97%—initiate 5-ASA treatment within the first year following diagnosis and a substantial proportion (60–87%) maintain therapy over the following decade [15]. Various 5-ASA formulations are available, all demonstrating comparable efficacy and safety, allowing flexibility in clinical use [8]. Since its introduction in 1942, sulfasalazine (SASP) has significantly enhanced the therapeutic outcomes in the management of UC [16]. Sulfasalazine is composed of 5-ASA, the active therapeutic component, bound to sulfapyridine, which serves as a carrier. This structure enables the drug to bypass absorption in the upper gastrointestinal tract and reach the colon, where colonic bacteria cleave it to release 5-ASA locally at the site of inflammation. Following metabolism, the components are excreted via the kidneys and bile. Its mechanism of targeted delivery underpins its therapeutic efficacy and its use continues to be evaluated alongside other pharmacological options to optimize UC management [16,17,18]. Since the adverse effects of SASP are largely attributed to its sulfapyridine component, newer salicylate-based drugs with improved safety profiles, free of sulfapyridine, have been developed and recently introduced for the treatment of UC [17,19]. Due to intolerance to the sulfapyridine component of SASP, some patients may experience gastrointestinal symptoms such as nausea, vomiting, dyspepsia, appetite loss or headaches. However, approximately 80% of those affected can tolerate alternative oral 5-ASA formulations that lack this moiety [13]. These newer agents, such as mesalazine, offer a better safety profile and act locally on the colonic mucosa to exert their anti-inflammatory effects.

**Figure 1:**
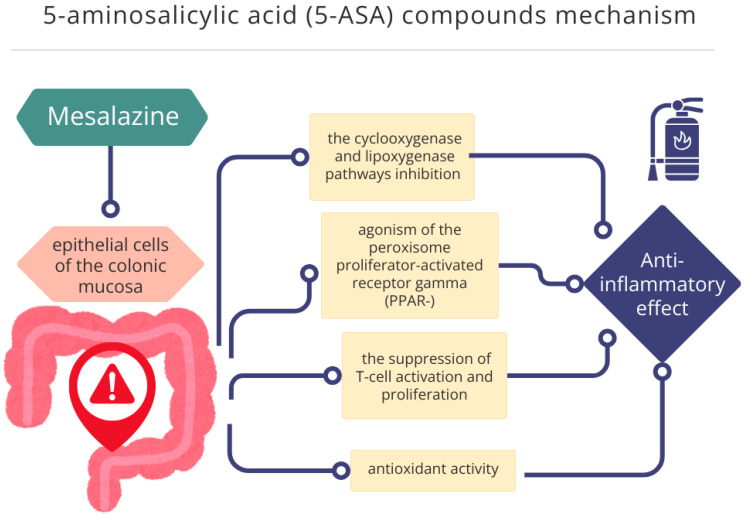
Summary of mechanism of action for 5-aminosalicylic acid (5-ASA) compounds.

Mesalazine is an anti-inflammatory agent with a complex, yet not fully elucidated mechanism of action in UC [20]. Its therapeutic effect is primarily local, targeting the epithelial cells of the colonic mucosa and modulating various inflammatory pathways [21]. Mesalazine is thought to inhibit the cyclooxygenase and lipoxygenase pathways, thereby reducing the synthesis of pro-inflammatory mediators such as prostaglandins and leukotrienes [22]. A key component of its activity involves agonism of the peroxisome proliferator-activated receptor gamma (PPAR-), which plays a critical role in regulating intestinal inflammation. Additionally, mesalazine may exert antioxidant effects and contribute to the suppression of T-cell activation and proliferation, further supporting its anti-inflammatory properties [20,22]. Various oral 5-ASA formulations have been developed to optimize drug delivery to the inflamed colonic mucosa while minimizing systemic side effects [20]. Following this discovery, new 5-ASA formulations were developed to limit small bowel absorption and enhance colonic delivery [19]. These include pH-dependent and time-dependent release systems, such as Eudragit-coated tablets, extended-release microgranules and multimatrix formulation (MMX) technology, which ensures availability throughout the colon. Such advancements allow for targeted therapy and improved patients’ adherence, particularly with once-daily dosing options [20,22]. Following the development of oral formulations, rectal mesalazine preparations such as suppositories and enemas have been proven to be highly effective, especially in patients with distal colitis. Studies consistently demonstrate that the continuation of topical therapy, combined with oral 5-ASA leads to significantly lower relapse rates compared to oral treatment alone, supporting their role in maintaining remission [16,23,24]. The mechanism of action for 5-ASA formulations is presented in Figure 1 below.

### 2.2. Moderate to Severe Activity—Glucocorticoids

The widespread use of glucocorticoids (GC) in many fields of medicine is primarily due to their multi-level action, including strong anti-inflammatory and immunosuppressive activity [25].

Glucocorticoids are steroid hormones, which gives them lipophilic properties, allowing them to easily diffuse across the cell membrane and bind to glucocorticoid receptors (GR) in the cytosol. Upon binding to a steroid molecule, the initially inactive GR receptor forms a GR/GC complex [26]. By increasing the expression of anti-inflammatory genes while simultaneously reducing the expression of pro-inflammatory ones, the GR/GC complex is responsible for anti-inflammatory and immunosuppressive effects, it also reduces the functioning of pro-inflammatory factors, including nuclear factor kappa-light-chain-enhancer of activated B cells (NF-κB) and activator protein 1 (AP-1) [25,26].

The non-specific, non-genomic mechanism of action of GCs is related to their immunosuppressive activity, but most of them remain unexplained.

Overall [25,26], the mechanism of action of glucocorticoids allows for the control of the inflammatory process in the large intestine and modulation of the immune response. In the context of IBD, reducing the expression of adhesion molecules plays a key role, which can limit the migration of inflammatory cells to diseased sites [27]. In patients with moderate to severe UC, it is often necessary to intensify treatment with GCs. The most common treatment option in these situations is oral prednisone at a dose of 40 mg daily. If more rapid intervention is necessary or the patient’s clinical condition is severe, intravenous steroids, such as hydrocortisone at a dose of 300 mg and methylprednisolone (60 mg), are warranted [13].

Prednisone is the most frequently chosen medication for the treatment of inflammatory conditions, not only in the gastrointestinal tract. It is a cortisone derivative with anti-inflammatory and immunosuppressive properties. Prednisone is a prodrug that, when activated by the enzyme 11β-hydroxysteroid dehydrogenase (11β-HSD), is converted to prednisolone, which exerts steroid-like pharmacological effects [26]. Furthermore, by inhibiting vascular permeability, it reduces swelling and congestion, which is an important element of UC treatment. Glycemic control should be monitored when using prednisone, as it increases protein catabolism and gluconeogenesis, which can lead to increased blood sugar levels. Prednisone also requires monitoring of the electrolyte profile, as it increases potassium excretion and increases sodium and water retention, which can manifest as edema, particularly in the lower limbs and face. Patients taking GCs should be advised to supplement calcium to prevent osteoporosis, as prednisone inhibits calcium absorption from the gastrointestinal tract [28]. Prednisone doses are typically 0.1–1 mg/kg of body weight, which typically amounts to approximately 40 mg per day. Methylprednisone is dosed similarly due to its comparable anti-inflammatory properties. The advantage of using both medications is their rapid onset of action, which is crucial in cases of UC flares. Induction therapy typically lasts 2–4 weeks and allows most patients to achieve symptomatic remission. The entire course of corticosteroid treatment should not exceed 8–12 weeks to minimize the risk of side effects typical of systemic steroids [29], such as fluid retention, weight gain, increased risk of infection, hypertension, hyperglycemia, and changes in lipid profile [26]. Oral beclomethasone dipropionate is a second-generation corticosteroid and may also be an alternative to conventional corticosteroids [30]. This formulation is also enteric-coated, enabling delivery of the active pharmaceutical ingredients (API) directly to the distal small intestine and colon [27]. A randomized, controlled trial of 282 patients proved that oral beclomethasone dipropionate at a dose of 5 mg daily for 4 weeks, followed by weekly dosing for another 4 weeks, demonstrated similar efficacy to prednisolone, without causing as severe side effects, and maintaining morning cortisol levels below 150 nmol/L. In patients with active left-sided or extensive UC, oral beclomethasone dipropionate at a dose of 5 mg/day has been shown to be equivalent to 2.4 g of 5-ASA and is more effective when added to 5-ASA compared to 5-ASA alone [30].

Budesonide is a synthetic glucocorticoid with potent local anti-inflammatory effects, particularly valued in the treatment of left-sided UC [29]. Structurally, it is like prednisolone but has a 15-fold higher affinity for the glucocorticoid receptor and significantly greater local activity [31]. Its chemical structure contains asymmetric 16α and 17α acetyl groups, which allows for the formation of a mixture of two isomers: 22R and 22S. Both epimers exhibit biological activity and a similar half-life, but the 22R form is approximately 2 to 3 times more potent than its counterpart [32]. It is currently available in several oral forms: a controlled-release formulation (CIR) in the colon, in which the release of the active substance is time- and pH-dependent, a time-dependent release formulation, and a MMX [31]. Another aspect supporting the use of budesonide is its high first-pass effect (over 90%), which minimizes its side effects and makes it better tolerated than traditional steroids [29]. Budesonide, in the form of MMX, ensures that the tablet structure remains intact in the stomach and small intestine [33], which translates into controlled and systematic release of the drug in the large intestine, which is an important aspect of effective therapy. The drug is available in Poland at a dose of 9 mg/day orally. Therapy typically lasts 8 weeks and, unlike traditional corticosteroids, does not require gradual withdrawal. The best results with budesonide have been documented in patients with left-sided colitis [29]. Rectal forms of budesonide, such as enemas or foams, have well-documented efficacy, particularly appreciated in left-sided UC [32]. Budesonide, in addition to minimizing the risk of side effects, does not cause significant adrenal suppression compared to traditional glucocorticoids (33% and 55%, respectively) [30]. It is a second-generation glucocorticoid, which means it undergoes a strong first-pass effect in the liver, leading to reduced systemic bioavailability [27]. The choice between topical and systemic preparations depends on the severity of disease symptoms and the patient’s clinical condition. Budesonide appears to be the preferred treatment for less active disease, where local action is sufficient with continued maintenance therapy with mesalazine. In cases of increased symptom severity and ineffective 5-ASA therapy, prednisone or methylprednisolone should be initiated. Their faster and more intense action is essential in more severe UC exacerbations [29]. If patients require long-term oral steroid therapy, it is recommended to monitor blood pressure, blood glucose, and potassium levels, and recommend bone densitometry to assess the risk of osteoporosis [34]. Regardless of the chosen treatment, it is important to monitor patients for side effects or the need for treatment modifications [29]. The occurrence of glucocorticoid resistance in IBD is a major problem, affecting up to 30% of patients. Research on impaired sensitivity to glucocorticoid inhibition in IBD, including UC, has focused primarily on three potential mechanisms. The first is overexpression of P-glycoprotein, which causes reduced cytoplasmic glucocorticoid concentrations secondary to increased glucocorticoid efflux from target cells. The second cause of resistance is increased expression of a truncated splice variant of the normal receptor isoform (GR), which does not bind glucocorticoid ligands. The third cause is the continuous activation of proinflammatory mediators NF-κB, AP-1, p38 protein kinases, and c-Jun N-terminal kinase (JNK). This functional interference may inhibit anti-inflammatory effects by preventing transcriptional activity [35,36].

### 2.3. Thiopurines in the Treatment of Steroid-Refractory Ulcerative Colitis

In the case of steroid resistance, i.e., a lack of clinical improvement during induction therapy, or steroid dependence—a clinical remission during steroid therapy, but a flare during dose reduction or within 3 months of discontinuing treatment—next-line medications are used. Thiopurines are the next step and can be used exceptionally in moderate to severe UC. A disadvantage of this group of drugs is that long-term use is required to achieve optimal therapeutic effect, which is not recommended if rapid clinical improvement is required [29].

**Figure 2:**
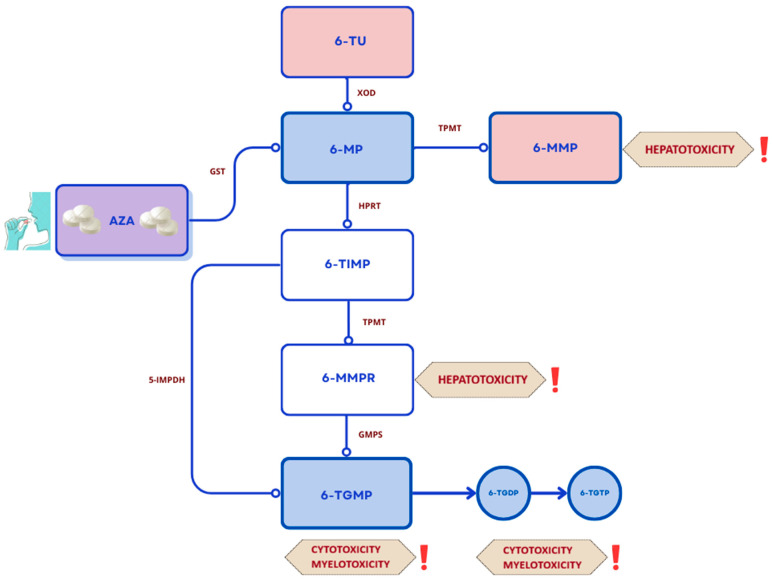
Thiopurine metabolism after oral administration of azathioprine (AZA). 6-MP—6-mercaptopurine; XOD—xanthine oxidase/dehydrogenase; 6-TU—6-thiouric acid; TPMT—thiopurine methyltransferase; 6-MMP—6-methylmercaptopurine; HPRT—hypoxanthine phosphoribosyltransferase; 6-TIMP—6-thioinosine monophosphate; 5-IMPDH—inosine 5′ monophosphate dehydrogenase; 6-MMPR—6-methylmercaptopurine ribonucleotide; GMPS—guanosine monophosphate synthetase; 6-TGMP—6-thioguanosine monophosphate; 6-TGDP—6-thio-guanosine diphosphate; 6-TGTP—6-thioguanosine triphosphate.

After oral administration, thiopurine derivatives are metabolized by several enzymes (Figure 2).

Thiopurines used in the treatment of UC include azathioprine (AZA) and 6-mercaptopurine (6-MP). AZA is a prodrug that, after undergoing complex metabolic processes, is converted to the pharmacologically active nucleotide 6-thioguanine (6-TGN). In the absence of enzymes or by the action of the enzyme glutathione S-transferase, azathioprine is converted to 6-mercaptopurine. 6-MP is metabolized by three competing enzymes: xanthine oxidase (XO), thiopurine-S-methyltransferase (TPMT), and hypoxanthine phosphoribosyltransferase (HPRT), which convert it to thiourea acid (6-TUA), 6-methylmercaptopurine (6-MMP), and precursors of active 6-TGN [37]. The immunosuppressive effects attributed to thiopurines occur through several mechanisms, the most common of which include inhibition of the expression of proinflammatory genes, such as *ITGA4* and tumor necrosis factors [38]. Other researchers argue that a key effect of azathioprine is the inhibition of Rac1 GTPase activation by the AZA metabolite 6-thioguanine triphosphate (6-Thio-GTP) in human CD4+ T lymphocytes [39]. AZA (at a dose of 2–2.5 mg/kg body weight) or 6-MP (at a dose of 1–1.5 mg/kg body weight) is administered orally in one or two divided doses. Due to the long time required to achieve a therapeutic effect (6–12 weeks), the use of thiopurines during the remission induction phase is not recommended. Consideration can only be given to maintaining steroid therapy at the lowest effective dose to control symptoms and then attempting to discontinue them. Treatment with AZA and 6-MP is associated with a relatively high risk of adverse effects [29]. These include leukopenia, bone marrow suppression, hepatotoxicity and kidney damage, gastric disorders, and pancreatitis. Long-term therapy has been associated with an increased risk of non-melanoma skin cancer, lymphoma, and cervical cancer [40]. Due to the increased risk of adverse events during thiopurine therapy, clinical monitoring and regular laboratory tests—including complete blood counts, AST and ALT levels, and creatinine—are mandatory every two weeks for the first two months of treatment, and then at least every three months [38]. To monitor thiopurine therapy, monitor for side effects, and in the event of an incomplete response to treatment, it may be helpful to measure the concentrations of the active metabolite 6-TGN and 6-MMP (the metabolite associated with the most side effects) in red blood cells. Low 6-TGN concentrations may indicate inappropriate drug administration or the need to optimize the dosing regimen. Normal levels, however, may suggest a change in therapy or a switch to a drug with higher therapeutic potential. Furthermore, before initiating therapy with thiopurine derivatives, it is recommended to measure thiopurine methyltransferase activity—a low result or no activity will contraindicate treatment [29]. It is worth mentioning the beneficial effect of thiopurines in the context of protection against colon removal. A more than 20-year study in which thiopurine therapy lasted more than 12 months observed more than 70% reduction in the risk of colectomy in patients with late-onset UC [40].

Conventional treatment options for UC are summarized in Table 1.

## 3. Biological Treatment

### 3.1. Infliximab

Infliximab (IFX) is the most widely and longest-used biologic drug, belonging to the group of tumor necrosis factor alpha (TNF-α) inhibitors [41]. It is a chimeric monoclonal antibody containing a murine Fab fragment (25%) and a human Fc fragment (75%). It binds both soluble and insoluble forms of TNF-α with high efficacy, thus inhibiting their proinflammatory effects [42]. The mechanism of action of IFX in the context of IBD focuses on reducing the expression of cytokines and inflammatory mediators in intestinal tissues. TNF is extensively produced by immune cells during the inflammatory process and then participates in their migration from blood vessels to diseased sites. By inhibiting TNF with IFX, it is possible to control the symptoms of UC [43]. Furthermore, this antibody has been shown to induce the lysis of membrane-bound TNF-alpha (mTNF-α)—expressing cells through complement activation or cytotoxicity. Furthermore, infliximab stimulated apoptosis in monocytes and T lymphocytes in the lamina propria of the large intestine [44]. Matrix metalloproteinases (MMPs) and their tissue inhibitors (TIMPs) are considered fundamental to maintaining control of physiological processes in the intestines and are produced by numerous cells in the gastrointestinal tract, including T lymphocytes, monocytes, macrophages, and mesenchymal cells. Infliximab therapy helped maintain the balance between MMPs and TIMPs, which is often disturbed in inflammatory bowel disease. Furthermore, the ability of IFX to induce healing of the colonic mucosa was examined. Analyses showed that induced regulatory macrophages were responsible for the endoscopic improvement, indicating that they play a key role in repair processes, and IFX possesses a component that activates the activity of these cells [43,45]. Considering method of drug administration, IFX is the most used biologic drug in clinical practice in Poland. The treatment regimen involves intravenous administration of the drug at a dose of 5 mg/kg body weight at weeks 0, 2, and 6 during induction therapy, and then every 8 weeks during maintenance therapy. The subcutaneous formulation of IFX has been available in Poland since December 2022, after positive opinion issued by the Committee for Medicinal Products for Human Use (CHMP) of the European Medicines Agency (EMA) in June 2020. The Food and Drug Administration (FDA) approval was issued later in 2023 for infliximab-dyyb. The advantages of this antibody undoubtedly include a rapid and potent onset of action, a favorable safety profile, and relatively high immunogenicity [29]. IFX, like any other drug, is associated with side effects, but those reported so far have been mild. These most commonly include headaches, increased incidence of upper respiratory tract infections, and skin reactions [42]. Hypersensitivity reactions during drug infusion and leukopenia have also been reported [43]. A slight increased risk of skin melanoma has also been suggested. As a biologic, IFX poses a risk of developing antiantibodies, which could reduce the effectiveness of the therapy. Therefore, thiopurine therapy should be considered within the first year of treatment to minimize this phenomenon [29]. IFX was the leading agent in terms of its efficacy in inducing clinical remission and endoscopic improvement in biologic-naive UC patients [46].

### 3.2. Adalimumab

Adalimumab (ADA) is another drug belonging to the TNF inhibitor class, this fully human recombinant IgG1 antibody is involved in inhibiting neutrophil activation and leukocyte migration to sites of inflammation. By interacting with TNF, adalimumab induces antibody-dependent cytotoxicity, stimulates the complement system, and induces T-cell apoptosis [47]. ADA is administered subcutaneously at a dose regimen of 160-80-40 mg every two weeks, with induction therapy lasting 12 weeks. In addition to lower immunogenicity, it has similar properties to IFX, thus substituting medications may be considered if efficacy is lost. During both ADA and IFX treatment, regular monitoring of serum concentrations of these drugs and, if necessary, determination of neutralizing antibody levels is recommended, which may be helpful in personalizing therapy [29]. Studies comparing the rate of healing of the colonic mucosa in patients undergoing therapy with ADA and IFX have not revealed significant differences. Therefore, it can be concluded that they are similarly effective [41]. However, the recent meta-analysis led by Salman, stated that even after comparable efficacy, the IFX may be the preferred first-line treatment due to its strong induction phase effect. Furthermore, the choice between IFX and ADA should be made on an individual basis, considering comprehensive clinical evaluation and patient specific factors [48].

### 3.3. Vedolizumab

Vedolizumab is a humanized monoclonal antibody that aims to limit lymphocyte migration toward the intestines by specifically binding to the α4β7 heterodimer found on the surface of intestinal lymphocytes. Compared to natalizumab, this antibody does not affect lymphocyte migration to the brain [49]. Vedolizumab’s mechanism of action is primarily based on disrupting the transport of T cells and leukocytes, which involves the adhesion, activation, and migration of these molecules across vascular walls. These processes trigger the release of several proinflammatory cytokines, which, by stimulating endothelial cells, increase the expression of adhesion molecules from the integrin family and activate the recruitment of proinflammatory cells. T-cell migration is mediated by integrin 1 associated with leukocyte function (LFA-1 or α2β2) and two α4 integrins (α4β1, α4β7), which interact with specific endothelial ligands, i.e., addressins, including vascular cell adhesion molecule 1 (VCAM-1), mucosal vascular addressin cell adhesion molecule 1 (MAdCAM-1), and intercellular adhesion molecule 1 (ICAM-1). In the context of the intestine, integrin α4β7 plays the most important role, as it is present in an intestinal lymphoid tissue and binds to MAdCAM-1. The remaining integrins—α4β1 and α2β2—interact with VCAM-1 and ICAM-1 [50]. Vedolizumab interacts exclusively with the gut-specific integrin α4β7; it does not bind to any other heterodimers containing α4 and β7, including α4β1 and αEβ7. This lack of binding activity excludes adverse effects outside the gastrointestinal tract, including in the central nervous system. Vedolizumab’s selectivity for gastrointestinal mucosa has been confirmed in numerous clinical studies [51]. During induction therapy, vedolizumab is administered at a dose of 300 mg as an intravenous infusion every 0, 2 and 6 weeks. During maintenance therapy, the patient receives the same dose every 8 weeks. If clinical need arises, treatment can be intensified with 300 mg every 4 weeks. Currently, a subcutaneous formulation of vedolizumab containing 108 mg is also available, administered every 2 weeks by patients who have achieved clinical remission with intravenous infusions. Compared to IFX, vedolizumab has lower immunogenicity, which translates into a lower risk of serious infections and improved oncological safety [29]. The most frequently reported adverse events included exacerbation of UC, headaches, upper respiratory tract infections and nasopharyngitis, nausea, and abdominal pain [52].

### 3.4. Etrolizumab

Etrolizumab is a new-generation anti-adhesion molecule. It is a humanized monoclonal antibody that selectively binds to the β7 subunit, which is part of the heterodimers of both α4β7 and αEβ7 integrins [53]. Integrins are heterodimeric proteins expressed on the surface of lymphocytes. They contain an outer membrane domain, acting as a receptor for various ligands, as well as transmembrane and intracellular domains, which participate in signal transduction. Initially, integrins are inactive; their activation occurs only after interaction with chemokines, which causes a conformational change and increases their affinity for ligands [54]. Integrin α4β7 plays a significant role in leukocyte infiltration into the gastrointestinal tract by interacting with MAdCAM-1 in the vascular endothelium of the mucosa. Additionally, it selectively binds to E-cadherin, which favorably influences T-cell adhesion to the intestinal epithelium. Therefore, integrin α4β7 and E-cadherin have become new therapeutic targets due to their elevated levels found in colon tissue from patients with IBD [55]. Etrolizumab has a bimodal mechanism of action, corresponding to two therapeutic targets [53]. This antibody can inhibit leukocyte migration into the intestine by inhibiting α4β7 and MAdCAM-1 and retain leukocytes in the intestinal epithelium by blocking the interaction between E-cadherin and αEβ7. This binding is thought to reduce lymphocyte adhesion and retention, potentially reducing inflammation in the intestine. Supporting etrolizumab’s selectivity, it has been shown that etrolizumab does not participate in the α4β1+/α4β7− interaction with VCAM-1, even at very high therapeutic concentrations. Furthermore, in animal studies, blocking β7 integrin prevented T-cell migration into the inflamed intestine, contributing to a better understanding of the drug mechanism of action [54,55]. Etrolizumab is characterized by low immunogenicity [56], but there is no clear data on which dose is most effective [55]. In one study, a single dose was modified by gradually increasing it. The following doses were administered: 0.3 mg/kg intravenously; 1.0 mg/kg intravenously; 3.0 mg/kg intravenously; 10 mg/kg intravenously; and 3.0 mg/kg subcutaneously [53]. Etrolizumab was generally well tolerated and safe in patients with UC. Since it does not involve the mechanism of leukocyte infiltration into the nervous system, its use minimizes the occurrence of progressive multifocal leukoencephalopathy (PML) [55]. The most serious adverse event reported while taking etrolizumab was a flare of UC. Other side effects were mild and did not require treatment discontinuation, including headache, dizziness, abdominal pain, chronic fatigue, nausea, nasopharyngitis, and urinary tract infections [57]. Previously published data regarding phase 2 induction study demonstrated that etrolizumab has a beneficial effect on reducing inflammatory biomarkers and achieving clinical, endoscopic, and histological remission. However, there is still a lack of research to solidify this drug’s position in the treatment of IBD, especially after mixed results achieved in phase 3 study (LAUREL), where contrary to earlier findings, no significant differences were observed between etrolizumab maintenance treatment and placebo for the primary endpoint of remission [58]. Perhaps a comparative analysis with other drugs with a similar mechanism of action, such as vedolizumab, would contribute to a better assessment of efficacy and safety and aid in selecting the optimal, personalized therapy for a specific patient [56].

### 3.5. Golimumab

Golimumab (GLM) is a fully human monoclonal antibody that forms stable complexes with high affinity for TNF, enabling this factor to bind to its receptors [59]. GLM is the latest addition to anti-TNF therapy. It is distinguished primarily by its degree of binding to tumor necrosis factor and protein stability. Studies have shown that it has a higher affinity for both soluble and transmembrane TNF and, compared to IFX and ADA, neutralizes TNF more effectively. Additionally, GLM is characterized by higher conformational stability and a better ability to inhibit TNF-induced cytotoxicity and endothelial cell activation [60]. The main target in the mechanism of action of golimumab is binding to human TNF and neutralizing the expression of adhesion molecules, including E-selectin, VCAM-1, and ICAM-1, on the surface of vascular endothelium. *In vitro* studies have demonstrated an inhibitory effect of GLM on the secretion of IL-6, IL-8, and granulocyte-macrophage colony-stimulating factor (GM-CSF), stimulated by TNF. Patients taking GLM benefited from the therapy by lowering their CRP levels, which translated into significant reductions in other parameters: IL-6, ICAM-1, MMP-3, and VEGF levels [61]. GLM is available as a 0.5 mL and 1 mL solution, corresponding to 50 mg and 100 mg of the active ingredient, respectively, in a single-dose prefilled syringe or an autoinjector for subcutaneous administration [62]. Intravenous administration of GLM did not produce the expected clinical results [63]. The standard dosing regimen for UC is 200 mg, 100 mg, and 50 mg at weeks 0 and 2, respectively, followed by the lowest dose every 4 weeks. Four-week dosing intervals for GLM are possible due to its significantly higher affinity for soluble human TNF-α [62]. Currently, there is no data on combining GLM with immunomodulators in the treatment of UC. There are also no studies evaluating the efficacy of combining GLM with azathioprine, so the efficacy and safety profile of combined therapy cannot be determined [63]. The most reported adverse events include post-infusion reactions, such as headache, rash, and fever. Less common adverse events include serious skin reactions, cellulitis, and bacterial infections. Other symptoms include an increased risk of lymphoma, demyelination, and meningitis [34]. In summary, there are no significant differences in the induction of clinical remission in patients treated with IFX, ADA, or GLM. Comparing the safety profile of GLM with other anti-TNF agents did not reveal any significant differences [63].

### 3.6. Ustekinumab

Ustekinumab is another human monoclonal antibody that blocks IL-12 and IL-23 [64] and targets the p40 subunit of both these interleukins [65]. Its mechanism of action is based on binding to the p40 subunit of IL-12 and IL-23, which inhibits interaction with the IL-12 receptor, its signaling, and subsequent activation of Th1 cells. Inhibition of Th17 cell activity occurs by affecting the IL-23-dependent immune response [66]. For the induction of remission in UC, this drug is administered intravenously based on the patient’s body weight, and then, during continued therapy, the drug is switched to a subcutaneous formulation, which can be administered at a dose of 90 mg every 4, 8, or 12 weeks, depending on the response to treatment [29,67]. Studies have reported that treatment with ustekinumab led to a significant reduction in inflammatory biomarkers, including CRP and calprotectin, as early as week 2 of therapy [65]. Ustekinumab was found to be well-tolerated by patients with UC, and the adverse event profile did not differ significantly from previously known medications. Serious opportunistic infections were very rare and no delayed hypersensitivity reactions were reported [68]. In summary, ustekinumab is characterized by low immunogenicity and a favorable safety profile [29].

### 3.7. Other Drugs Targeting IL-12/IL-23

Research shows that the interaction between IL-12 and IL-23 has a significant impact on maintaining intestinal homeostasis. IL-12 activates the Th1 pathway, which includes the cytokines IFNγ and TNF, and stimulates group 1 lymphoid cells (ILCs). IL-23, in turn, influences the Th1 pathway and stimulates ILC3 and NKT cells. The effects of these interactions have been confirmed as causative factors in promoting intestinal neoplasia, a complication of chronic intestinal inflammation. Drugs targeting IL-12/IL-23 are currently in intensive clinical development, with guselkumab and mirikizumab being the most frequently mentioned [69].

#### 3.7.1. Guselkumab

Guselkumab is a human IgG1 monoclonal antibody directed against the p19 subunit of interleukin-23 [70], currently approved for the treatment of psoriatic arthritis and plaque psoriasis [71]. The currently ongoing phase 3 double-blind, randomised, placebo-controlled induction and maintenance studies in patients with UC (QUASAR) are aimed at evaluating the efficacy and safety of guselkumab. The first results are promising, a significantly higher proportion of patients treated with guselkumab achieved clinical remission at week 12 of induction (adjusted treatment difference 15%) and at week 44 of maintenance treatment (adjusted treatment difference 25–30%) with overall favorable safety profile [72].

#### 3.7.2. Mirikizumab

Mirikizumab is a humanized variant of an IgG4 monoclonal antibody that demonstrates selectivity for the p13 subunit of interleukin-23. The confirmed efficacy of IL-12/IL-23 blockade with mirikizumab represents a promising advance in the development of selective treatment. Visible clinical and endoscopic improvement was observed after just 4 weeks of therapy, which is particularly important in more severe forms of the disease [70]. Regarding the safety of therapy with anti-IL-12/IL-23 drugs, most adverse events in randomized controlled trials were mild and did not require treatment discontinuation [69]. The clinical usefulness and safety were confirmed during the multicenter, parallel-group phase 3 LUCENT-1 and LUCENT-2 clinical trials, which compared mirikizumab with placebo in patients with moderately to severely active UC. Results indicated that mirikizumab led to higher clinical response rates at week 12 (induction phase LUCENT-1) among the overall study population, among biologic-naive patients, and among biologic-experienced patients. Similar efficacy was achieved in maintenance phase (40 weeks, LUCENT-2) across clinical, symptomatic, endoscopic, and histologic measures of disease. What is more, even in patients with treatment failure (conventional immunosuppressive agents, biologic therapies, or tofacitinib) [73]. This further led to FDA and EMA approval (2023) for mirikizumab as a subcutaneous, single-injection, once-monthly maintenance regimen for adults with moderate to severe UC and remains as the first approved IL-23p19 inhibitor for this disease.

Summarizing the currently available data, there are no clear conclusions as to whether IL-23 blockade yields better results than TNF blockade. The greatest advantage of IL-23 inhibitors appears to be their good safety profile, which also opens the possibility of combining them with other drugs with different mechanisms of action, such as anti-integrin antibodies. From a pathophysiological perspective, combining anti-IL-23 with anti-TNF may be an interesting prospect, as available data suggest that resistance to anti-TNF treatment may result from excessive activation of the IL-23 pathway. Studies are ongoing to evaluate this phenomenon using the combination of guselkumab with golimumab [74]. Moreover, the phase 3 LUCENT-3 clinical trial is ongoing, with completion date estimated at the end of 2027, which aims to evaluate the long-term efficacy and safety of mirikizumab in participants with moderately to severely active UC. The first reports confirm the effectiveness and safety following 152 weeks of continuous treatment, giving hope for the widespread use of this inhibitor in the near future [75].

**Figure 3:**
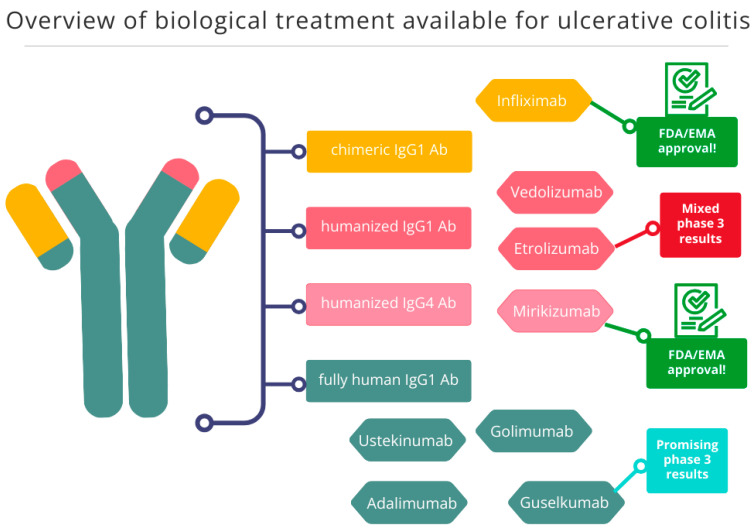
Graphical presentation of available antibodies for the treatment of ulcerative colitis.

The summary of biological treatment in UC is presented below in Table 2 and Figure 3.

## 4. Other Drugs and Experimental Agents Used in Treatment of Ulcerative Colitis

### 4.1. JAK Inhibitors and Immunomodulators

JAK (Janus activated kinase) inhibitors are gaining increasing popularity in the treatment of IBD and many other autoimmune conditions. Compared to biologics, these small-molecule drugs are characterized by a rapid onset of action, a short half-life, and a low risk of immunogenicity. Moreover, they can be administered orally [76]. The janus kinase-signal transducer and activator of transcription protein (JAK/STAT) pathway is composed of four components: JAK1, JAK2, JAK3, and tyrosine kinase 2 (TYK2), which play a crucial role in the signaling of a few proinflammatory cytokines that predispose to the development of inflammatory bowel disease. JAK activation occurs only after the cytokine binds to its receptor, which leads to the phosphorylation of STAT proteins. Only then, through various cellular signals, can STATs generate an inflammatory response induced by specific cytokines, including IL-6, IL-9, IL-12/23, and IFN-γ. Inhibition of the JAK/STAT pathway appears to be a promising treatment option for UC and Crohn’s disease, as it helps minimize chronic inflammation in the intestines [77]. Compared to TNF inhibitors and α4β7 integrin inhibitors, which block single or several specific molecules, JAK inhibitors are able to simultaneously inhibit multiple cytokines at the level of different cell signaling pathways, predisposing to more effective therapy. Furthermore, JAK inhibitors do not induce the development of anti-drug antibodies, minimizing immunogenicity. Furthermore, the effect of inhibiting a specific JAK kinase is associated with a unique mechanism of action, which creates extensive opportunities for personalized therapy [78].

#### 4.1.1. Tofacitinib

Tofacitinib was the first FDA- and EMA-approved small-molecule drug that non-selectively inhibits Janus kinases, intended for the treatment of moderate to severe UC [29,78]. It is used both in patients who have failed biological therapies and in those who have not previously received biological therapy [79]. *In vitro* studies have shown that tofacitinib preferentially inhibits JAK1 and JAK3, while having a weaker effect on JAK2 [80]. Currently, this drug is one of the most studied JAK inhibitors [77]. Tofacitinib is administered orally, with an initial dose of 10 mg twice daily for 8 weeks, and a maintenance dose of 5 mg twice daily. It has a rapid onset of action and a safety profile like IFX [29]. Available study results confirm the efficacy of tofacitinib in inducing clinical and endoscopic improvement in patients with UC [81]. Initial treatment improvements were visible after just 3 days of therapy, which was crucial for patients who had not achieved satisfactory results with first-line medications, such as intravenous glucocorticosteroids and immunosuppressive therapy [82]. Significant improvements in patients’ quality of life were also observed while taking tofacitinib, and these improvements remained high throughout the treatment period. Furthermore, the need for concomitant immunosuppressive therapy was not demonstrated, supporting the use of tofacitinib as monotherapy [81]. Reported adverse events were mild and did not differ significantly from placebo. However, patients receiving tofacitinib, both during remission induction and maintenance therapy, were observed to have an increased risk of infections, particularly *herpes zoster* [30]. The mechanism of this phenomenon remains unclear, but it is likely related to an impaired immune response to viral infections due to abnormalities in interferon communication and other signaling pathways involved in JAK/STAT inhibition. All reported skin symptoms associated with tofacitinib were mild and did not require treatment discontinuation. The nature of the lesions was concluded to be dose-dependent, but the incidence of skin lesions did not increase with continued treatment [81]. Furthermore, increased lipid levels of total cholesterol, HDL, and LDL have been observed with the drug, but no correlation has been demonstrated with increased cardiovascular risk [77]. However, caution is recommended in patients at increased risk of thromboembolic events [29]. Several serious adverse events, such as pneumonia, anal abscess, and *Clostridium difficile* infection, have been reported, but the incidence of these events was extremely rare [77]. Tofacitinib is a small molecule drug, therefore it does not carry the risk of inducing the production of neutralizing antibodies [29].

#### 4.1.2. Filgotinib

Filgotinib is a selective JAK inhibitor [83], demonstrating approximately 5-fold greater potency in inhibiting JAK1 compared to JAK2, JAK3, and TYK2 [54]. This drug is rapidly and efficiently absorbed from the gastrointestinal tract after oral administration and, *via* the carboxylesterase 2 isoform, is converted to a major metabolite that exhibits similar preferential activity for JAK1, but 10-fold lower biological activity. Furthermore, its systemic concentration is 16- to 20-fold higher compared to the parent compound [83]. The use of selective JAK inhibitors allows for dose reduction, which translates into minimized side effects while maintaining treatment efficacy [84]. This drug can also be used in steroid-dependent and steroid-resistant patients, as well as those who have not responded to immunosuppressive therapy. Another advantage of small-molecule drugs is the lower risk of hematological abnormalities compared to immunosuppressants, which require regular biochemical testing [54]. Filgotinib is administered orally at a dose of 200 mg daily, both for induction therapy (lasting 10 weeks) and for maintenance therapy [29]. The SELECTION clinical trial demonstrated that this dosage was well tolerated by patients, resulting in both clinical remission and its maintenance [85]. Analyses show that filgotinib is as effective as most drugs tested to date and has a beneficial effect on patients who have not previously received biological therapy [86]. Furthermore, endoscopic mucosal healing was similar to other therapies, including biologicals [87]. The most frequently reported adverse events during filgotinib treatment included rhinitis and headaches [54]. Compared to tofacitinib, the risk of *herpes zoster* infection was very low and did not correlate with dose [85]. Regarding interactions with other drugs, attention was paid to those metabolized by carboxylesterase 1 (CES1) and carboxylesterase 2 (CES2)—the main isoforms responsible for filgotinib metabolism. Among these, the following are listed: Fenofibrate, carvedilol, diltiazem, and simvastatin have been reported, as they may interfere with CES2 inhibition. However, *in vitro* studies have not demonstrated the clinical significance of these interactions. Similarly, no dose adjustment of filgotinib was considered necessary during treatment with P-glycoprotein inhibitors or inducers, CYP3A4 substrates, or proton pump inhibitors, as their effect on the drug pharmacokinetics was negligible. Furthermore, there is no evidence of a need for treatment modification in patients currently taking metformin, methotrexate, or oral hormonal contraceptives [83].

#### 4.1.3. Upadacitinib

Upadacitinib (UPA) is a selective JAK1 inhibitor. This class of drugs exhibits a characteristic pattern: the higher the dose, the lower the preferential activity for different JAK subtypes. This is due to differences in the concentrations required to achieve 50% maximum inhibition (IC50). UPA is most selective for JAK1, but at higher doses it can also inhibit JAK2 and, to a lesser extent, JAK3 and TYK2 [88]. Upadacitinib demonstrates over 100-fold higher biochemical selectivity for JAK1 compared to JAK3 and TYK2, and studies conducted on cell lines show that the preferential activity for JAK1 is approximately 60-fold greater than for JAK2. This selectivity for JAK1 differs from that of tofacitinib, which inhibits both JAK1 and JAK3, exhibits some activity on JAK2 and very limited activity against TYK2 [89]. Upadacitinib has also been used to treat other autoimmune diseases, including rheumatoid arthritis, psoriatic arthritis, and atopic dermatitis [90]. The efficacy of UPA in the treatment of UC has been demonstrated in studies during the ACHIEVE and U-ACCOMPLISH trials [29], including in patients who failed to achieve satisfactory results with glucocorticosteroids, immunosuppressive drugs, and biologics [91]. As part of induction therapy, UPA is administered orally at a dose of 45 mg for 8 weeks, followed by 30 mg or 15 mg for maintenance therapy. The induction period can be extended by another 8 weeks if clinically necessary [29]. For patients with hepatic impairment or reduced renal function, a reduction in the induction dose to 30 mg and the maintenance dose to 15 mg is recommended [92]. Patients have reported clinical improvement after just 2 weeks of UPA treatment [89]. Currently, there are no direct studies comparing upadacitinib with other advanced therapies for IBD. However, several network meta-analyses have been published comparing treatments with anti-TNF agents, integrin inhibitors, ustekinumab, tofacitinib, and filgotinib and evaluating them for efficacy and safety. Upadacitinib ranked first in terms of clinical response, achievement and maintenance of remission, and endoscopic improvement. These data were also confirmed in patients who had previously received other therapies, including biologics [92]. The most frequently reported adverse events during UPA therapy included acne, upper respiratory tract infections and nasopharyngitis, headaches, and increased creatine kinase levels [93,94]. Additionally, changes in lipid profiles were observed in patients treated long-term with UPA, including an increase in total cholesterol, while maintaining normal LDL and HDL cholesterol ratios. More cases of neutropenia were documented, but none of the above-described side effects required treatment discontinuation [92].

In summary, based on the available data, UPA appears to be an effective drug with a favorable safety profile. It appears to be a suitable treatment option for patients who, in addition to the active phase of UC, also struggle with musculoskeletal symptoms. Its route of administration, good tolerability, rapid clinical response, and efficacy against extraintestinal symptoms place UPA among the currently available drugs. Upadacitinib therapy also allows for consideration of individual patient needs and medical history, increasing the potential for personalized therapy [89,93].

All JAK inhibitors, whether non-selective (e.g., tofacitinib) or selective (filgotinib and upadacitinib), are contraindicated during pregnancy because they carry a risk of teratogenicity [54]. Despite the lack of data on the drug’s excretion into breast milk, UPA is not recommended for breastfeeding mothers [90,93].

### 4.2. Sphingosine-1-Phosphate Receptor Modulators

Sphingolipids are lipid compounds responsible for cell membrane function and maintaining proper cell shape. The skeleton of these molecules is composed of biologically inactive sphingosine, which is converted to sphingosine-1-phosphate (S1P) by sphingosine kinases. S1P activity is only noticeable after binding to the appropriate receptor—S1PR, which, depending on the isoform, performs a variety of functions, including maintaining the integrity of the epithelial barrier, supporting proper vascular tone, and participating in lymphocyte migration [94]. S1P levels are regulated by sphingosine phosphatase, which, through its presence in the gastrointestinal tract, catalyzes the dephosphorylation of S1P to free sphingosine. Elevated levels of this enzyme have been observed in models of intestinal inflammation, suggesting that its activity contributes to the pathomechanism of the disease [95]. Differences in S1P concentrations in blood, lymph, and lymph nodes regulate lymphocyte migration and participate in the activation of inflammatory molecules, including NF-κB and signal transducer and activator of transcription 3 (STAT3), which play a crucial role in cell proliferation and apoptosis [94]. After binding to its receptor, S1PR participates in numerous processes, such as adhesion, endocystosis, cell migration and proliferation, and T and B lymphocyte transport, which contributes to maintaining adequate vascular permeability. Furthermore, increased S1P expression has been shown to translate into increased levels of E-cadherin, an adhesion protein that strengthens the intestinal barrier, as confirmed in intestinal epithelial cell cultures [95]. Therefore, the use of S1PR modulators can mitigate the inflammatory response in IBD by binding lymphocytes in lymph nodes, which leads to a reduction in the pool of immune cells circulating in the blood [96]. In a properly functioning organism, low expression of S1P kinases and high S1P lyase (SPL) activity in the epithelium keep S1P concentrations at a negligible level. In the presence of inflammation, S1P concentrations increase dramatically due to the activation of sphingosine kinases. Studies conducted in mouse models of dextran sodium sulfate (DSS)-induced intestinal inflammation have shown that deletion of the *SphK1* gene resulted in a more severe disease course compared to the control group, while the severity of inflammatory changes was inversely correlated with S1P levels. Additionally, deletion of the *SPL* gene in mice resulted in increased levels of proinflammatory cytokines, resulting from S1P accumulation in the intestines, which in turn activated the STAT3 cell signaling pathway. Therefore, in recent years, S1P has received particular attention as a potential new therapeutic target due to its confirmed role in maintaining intestinal homeostasis and proper intestinal epithelial barrier integrity [94]. Furthermore, small molecule therapies offer patients not only the convenience of oral administration but also minimal risk of immunogenicity and a rapid onset of action [97].

#### 4.2.1. Ozanimod

Ozanimod is a potent S1P receptor modulator and exhibits high affinity and selectivity for the S1P1 and S1P5 subtypes, which are involved in regulating immune function [98]. It does not interact with, or only minimally interacts with, the S1P2, S1P3, and S1P4 receptors [99], eliminating the side effects associated with non-selective modulators [97]. *In vitro* studies showed that the two major metabolites of ozanimod demonstrated similar levels of activity and receptor selectivity compared to the parent compound [99]. The mean half-life of ozanimod is 21 h, while that of its two metabolites is approximately 11 days, which contributes to the maintenance of some of the drug’s effects even after treatment has ended. Therefore, for example, when planning a pregnancy, initiating immunosuppressive therapy, or administering a live vaccine, a 3-month treatment break is recommended after completing ozanimod therapy [97]. Clinical studies have demonstrated the efficacy of ozanimod in patients with UC in terms of clinical response, endoscopic improvement, and intestinal mucosal healing. Clinical and symptomatic remission was observed after just 10 weeks of treatment [100]. Partial clinical improvement based on the Mayo scale was observed after approximately 4–8 weeks of therapy, confirming the rapid onset of action of ozanimod, and these effects were maintained over time [95]. The frequency of bowel movements and rectal bleeding decreased starting from the second week of treatment [99]. Furthermore, this drug was observed to contribute to lower levels of inflammatory markers, including FC and lactoferrin, both in the induction phase and during maintenance treatment. Furthermore, the North study, among others, demonstrated that ozanimod significantly reduced the concentration of circulating neutrophils and the process of calprotectin degradation by the enzyme neutrophil elastase, which translates into a systemic reduction in inflammation [99]. The ozanimod dosing regimen is as follows: 0.23 mg orally for the first 4 days of treatment, followed by 0.46 mg for the next 3 days, and 0.92 mg chronically from day 8 onwards [29]. This drug demonstrated similar efficacy both as a stand-alone therapy and in combination with GCs, as well as in patients who did not respond to treatment with 5-ASA and monoclonal antibodies [99]. Ozanimod was well tolerated by patients, and reported adverse events were mostly mild and did not require treatment discontinuation. The most commonly reported side effects included upper respiratory tract infections, increased liver transaminase levels, orthostatic hypotension, urinary tract infections, back pain, hypertension, and upper abdominal pain [101]. The increased susceptibility to infections is a consequence of the drug mechanism of action, as it exerts immunosuppressive effects by sequestering lymphocytes responsible for the inflammatory process in the lymph nodes. However, it should be noted and the patient made aware that a decrease in lymphocyte counts is one of the expected therapeutic effects of ozanimod [97]. Furthermore, S1P signaling also affects cardiovascular function, so S1PR1 modulators, including ozanimod, may affect heart rhythm, such as bradycardia or atrioventricular block, which has been observed in studies, although the dose titration scheme effectively mitigates this effect, unlike the first-generation S1P modulators (e.g., Fingolimod). This drug may cause a transient decrease in heart rate, but it does not predispose to the development of clinically significant bradycardia or prolong the QT interval [99]. The effect of ozanimod on the cardiovascular safety profile stems from its lack of interaction with the S1P3 receptor, whose modulation is responsible for cardiac conduction abnormalities. Therefore, it is important to perform an electrocardiogram before initiating therapy to rule out potential abnormalities or detect existing ones. Additionally, it is recommended that patients taking certain medications that affect the cardiovascular system, such as β-blockers, calcium channel blockers (CCBs), antiarrhythmics, or QT-prolonging drugs, consult a cardiologist before starting treatment. Furthermore, S1P modulators may increase the risk of macular edema, so patients with risk factors, diabetes, or a history of uveitis should undergo an ophthalmological evaluation [97]. Among drug interactions, it is also worth noting that two of the main metabolites of ozanimod may have an inhibitory effect on monoamine oxidase-B, which translates into the accumulation of neurotransmitters metabolized by this enzyme, i.e., serotonin and norepinephrine. Therefore, concomitant use of SSRIs, SNRIs, and tricyclic antidepressants should be contraindicated due to the risk of hypertension. However, it is worth mentioning that analyses of clinical trial data did not confirm an increased incidence of hypertension with concomitant use of ozanimod and the aforementioned antidepressants. No cases of serotonin syndrome have been reported, so it appears that combining these drugs does not increase the incidence of adverse events in practice [98,102]. Significant contraindications to initiating ozanimod therapy include patients with cardiac comorbidities, liver failure, cancer, and pregnancy [29].

In summary, current studies show that ozanimod is highly effective, both as first-line therapy and in patients previously treated with other medications, including immunomodulators and biologics, and has a good safety profile. The effects of treatment were noticeable in terms of resolution of clinical symptoms, endoscopic improvement, and progress in intestinal mucosal healing, and were maintained over time [102].

#### 4.2.2. Etrasimod

Etrasimod is another sphycosin-1-phosphate receptor modulator designed to treat autoimmune inflammatory diseases [103]. It acts by interacting with the S1P1, S1P4, and S1P5 receptors, which is crucial in modulating the migration of immune cells, particularly lymphocytes, resulting in a reduction in their circulating pool. In the management of UC, etrasimod focuses on inhibiting the accumulation of immune cells within the colon [104]. It is a selective modulator, therefore activating only the S1P1, S1P4, and S1P5 receptors, without demonstrating activity against the S1P2 and S1P3 receptors. Unlike ozanimod, etrasimod does not require a gradual dose increase during therapy, as studies show that it is metabolized by various cytochrome P450 enzymes [105], minimizing the risk of potential delayed onset of action [106] and interactions with other drugs and food [105]. This drug also demonstrates advantages over ozanimod in terms of pharmacokinetics and metabolism. During the biotransformation of ozanimod, two metabolites with long biological half-lives are formed, significantly extending the time to first clinical effects. The half-life of etrasimod ranges from 29.7 to 36.7 h, and no potentially pharmacologically active metabolites have been detected [106], and the elimination half-life was approximately 1 week [105]. These aspects, especially its rapid onset of action, make etrasimod a favorite among S1P receptor modulators [106]. Etrasimod is administered orally at a dose of 2 mg daily. Besides confirming its efficacy and safety profile, studies have shown that this drug can rapidly reduce blood lymphocyte levels [107]. After just two weeks of therapy, patients’ lymphocyte counts were reduced by 50% compared to baseline values [105]. However, after discontinuing treatment, lymphocyte levels were observed to return to their initial values within approximately seven days [107]. Patients reported the first symptoms of remission after just two weeks of taking etrasimod, while endoscopic normalization was noticeable after approximately 12 weeks of treatment [105]. Etrasimod’s efficacy has been demonstrated both as monotherapy and in combination with glucocorticosteroids, and in patients previously treated with monoclonal antibodies or JAK inhibitors. This demonstrates that this drug is an effective method for achieving symptomatic, clinical, and endoscopic remission without the need for concurrent steroid therapy, which carries the risk of numerous side effects and has been an essential component of the treatment of severe UC [108]. The most frequently reported adverse events during therapy included increased susceptibility to infections, including upper respiratory tract and urinary tract infections, resulting from a decrease in blood lymphocyte counts, temporary bradycardia, increased hepatic aminotransferase levels, macular edema, and a transient increase in blood pressure. Side effects were mild and did not require treatment discontinuation. The only alarming symptoms associated with elevated liver function tests are nausea, vomiting, yellowing of the skin and whites of the eyes, and dark urine, which suggest consulting a physician to rule out liver damage [105,106,107]. In summary, treatment with etrasimod was characterized by good efficacy and a high safety profile, both in the induction and maintenance phases. It demonstrates several advantages related to oral administration and a rapid onset of action. Current studies show that this drug may be a promising new form of therapy for patients with UC, but additional information is still needed to optimize therapeutic strategies [105,107].

### 4.3. Toll-Like Receptor Modulators

Toll-Like Receptors (TLRs) participate in the recognition of pathogen-derived patterns (PAMPs) and damage-associated patterns (DAMPs) and constitute a component linking the innate and adaptive immune responses. Furthermore, depending on the activity and type of receptor, they can contribute to maintaining intestinal homeostasis on the one hand and activate the secretion of proinflammatory molecules on the other. Current research highlights the fact that abnormalities in the activation and expression of Toll-like receptors in intestinal epithelial cells, as well as *TLR* gene polymorphisms, may be involved in the pathogenesis and subsequent course of IBDs. Both TLR overexpression and reduced activity can have negative consequences in IBDs, including affecting the extent and severity of the inflammatory process [109,110]. Additionally, immunohistochemical evaluation revealed significantly increased expression of TLR2, TLR4, and TLR9 in the cytoplasm of epithelial cells from patients with UC compared to healthy individuals [111].

#### Cobitolimod

Cobitolimod belongs to the group of local TLR9 agonists, which, when activated within lymphocytes and antigen-presenting cells, leads to the stimulation of T-regulatory cells, increased IL-10 production, and inhibition of Th-17 [112]. Treatment with this drug has been observed to reduce the production of IL-12, known for its pro-inflammatory properties, and increase the production of IL-10, which contributes to the healing process of the intestinal mucosa [113]. Cobitalimod was administered as a rectal enema at three different doses: 31 mg, 125 mg, or 250 mg every three weeks or 125 mg every four weeks. Rectal administration of 250 mg was effective in inducing remission in patients with left-sided UC and was associated with a high safety profile and good tolerability [114]. Reported adverse events were similar in both the treatment and placebo groups and included headache, UC exacerbation, upper respiratory tract viral infections, and fever [112,114].

### 4.4. Adrenomodulin

Adrenomodulin (AM) is a biologically active peptide that was initially isolated from human pheochromocytoma. Functional AM receptors consist of the calcitonin-like receptor (CRLR) and the receptor activity-modifying protein (RAMP). Elevated plasma AM concentrations have been observed in patients with hypertension, heart failure, renal disease, or during the acute phase of stroke. Adrenomodulin is also detected in the gastrointestinal tract, particularly in the stomach and large intestine. Immunohistochemical analysis shows intense AM staining in tissues around gastric ulcers, and its concentration increases during healing and ulcer scarring. This may indicate that AM actively participates in cell healing and regeneration, and studies have demonstrated its beneficial effects on maintaining gastric blood flow, inhibiting gastric acid secretion, angiogenesis, and proliferation of epithelial cells in the mucosa [115,116]. The regenerative properties of adrenomodulin were utilized in a trial to treat UC. Following AM therapy, visible regeneration of the colonic mucosa and neovascularization were achieved. Ulcers disappeared within 8–12 weeks of treatment, and scarring appeared in their places. Patients receiving the drug reported only a decrease in blood pressure, but in none of the cases did this affect quality of life or constitute a clinical problem. Although AM therapy holds great promise, especially for patients with steroid-resistant UC, and the novel mechanism of action provides an additional favorable argument, further and more detailed studies are still needed to determine the optimal dosage and method of administration [117,118]. As of present this is an experimental agent and is currently not in standard UC treatment guidelines.

### 4.5. Apremilast

Apremilast belongs to the group of oral phosphodiesterase 4 (PDE4) inhibitors, which act intracellularly by modulating the activity of inflammatory mediators. By increasing the production of pro-inflammatory factors, including TNF-α and IL-23, and reducing the production of anti-inflammatory molecules, such as IL-10, the PDE4 enzyme participates in regulating the cellular response to inflammation. Apremilast blocks the synthesis of TNF-α and MMP3 in mucosal cells of patients with IBD. Currently, this drug is approved for the treatment of plaque psoriasis and oral ulcers associated with Behçet’s disease at a dose of 30 mg taken twice daily. Thus far, one study compared 30 mg and 40 mg doses of Apremilast with placebo in a randomized trial of patients with active UC. After 12 weeks of treatment, clinical remission was achieved in 32% of patients receiving 30 mg of apremilast. Numerical differences were also observed between the drug and placebo groups in clinically important variables, including reductions in inflammatory markers in stool and serum. Therefore, it is difficult to predict why statistically significant results were not achieved. In the study, the drug was well tolerated, did not predispose to an increased risk of infection, and the most frequently reported adverse event was headache. However, more research on the use of apremilast in the treatment of UC and its efficacy is needed, especially given its significant advantage of local rather than systemic action. As of present this is an experimental agent and is currently not in standard UC treatment guidelines [119].

### 4.6. Molecules That Increase miRNA Expression

Recently, therapeutics based on interactions with RNA have been gaining increasing interest. They are emerging as new therapeutic targets for diseases previously considered incurable, thanks to their functional and structural flexibility [120]. This experimental type of treatment may prove to be a lifesaver for patients who do not respond to monoclonal antibody therapy or who lose response over time. Furthermore, biological treatments are associated with a high risk of adverse effects, primarily related to immunogenicity. A more comprehensive understanding of disease pathogenesis is key to increasingly modernizing the treatment of nonspecific inflammatory diseases. Currently approved new targeted therapies, such as JAK inhibitors and S1PR modulators, although highly effective and with a good safety profile, may prove insufficient for patients with incomplete response or intolerance. Therefore, it is crucial to find new drugs from other molecular classes that will target additional mechanisms of disease development [121]. Dysregulation of miRNA expression correlates with the development of many neurodegenerative diseases and intestinal disorders, which is why attention has focused on the role of miRNAs in maintaining the proper functioning of the gut–brain axis. MiRNAs have the ability to regulate the expression of genes crucial for maintaining intestinal barrier integrity [122]. For example, miR-93 reduces the expression of tyrosine kinase 6 (*PTK6*), which activates claudin-3 expression, which influences the proper permeability of the intestinal epithelial barrier. Additionally, miRNAs play a significant role in regulating interactions between the gut microbiota and the immune system, which is crucial for maintaining homeostasis. It is worth mentioning miR-155, present in intestinal immune cells, which is responsible for controlling suppressor of cytokine signaling 1 (SOCS1) secretion, which helps maintain IFN-γ expression in NK cells at a constant level, which in turn reduces the activity of the nucleotide-binding domain, leucine-rich–containing family, pyrin domain–containing protein 3 (NLRP3) inflammasome, directly involved in the pathogenesis of UC. In the context of disease pathogenesis, miR-21, miR-155, and miR-31 are most frequently highlighted. Studies on animal models of intestinal inflammation induced by DSS or 2,4,6-Trinitrobenzene sulfonic acid (TNBS), the use of antibodies against miR-31 resulted in improved clinical outcomes. This is because miR-31 has the ability to inhibit the inflammatory response by regulating IL7R, IL17RA, and IL-25 levels and normalizing Th1/Th17 cell activity. Additionally, miR-155 influences cell membrane transport, and increased expression of miR-31 and miR-155 regulates the level of Il-13, one of the key cytokines in the pathogenesis of UC. Therefore, the use of a miR-155 inhibitor may support the functioning of the colonic mucosa, the layer to which the disease process is confined in UC. Despite the many potential benefits of using this group of molecules, only one preparation based on miRNA technology has been approved for clinical trials to date [116,118].

#### Obefazimod

Obefazimod (ABX464) is a synthetic aminoquinolone molecule that selectively increases the expression of miRNA-124, including in lymphatic cells, which contributes to the regulation of macrophage and monocyte activity and, consequently, reduces the concentration of proinflammatory proteins [121,123]. ABX464 binds to the cap-binding complex (CBC), located at the 5′ end of pre-mRNA, initiating several transcriptional and processing functions. It participates in the regulation of transcription factor secretion and efficient splicing [124]. Its pharmacological effect is the inhibition of the production of many key inflammatory mediators, including monocyte chemoattractant protein-1 (MCP-1/CCL2), the IL-6 receptor, and influences STAT3 and TNF-α converting enzyme (TACE) cell signaling [125]. The use of obefazimod in mice with DSS-induced colitis reduced the extent and intensity of inflammatory lesions in the colon and allowed the animals to return to their baseline body weight. These protective effects persisted over time despite continued exposure to DSS. Elevated levels of IL-22, a cytokine that plays a key role in tissue repair and inflammation, were also observed during therapy [121]. Obefazimod was originally studied for its ability to inhibit viral replication, and in this context, its efficacy was found in treating HIV infections, among other conditions [124]. Pharmacokinetic and toxicological analyses confirmed the rapid absorption of the obefazimod molecule, and the no-observed-adverse-effect level (NOAEL) was significantly higher than the dose required to inhibit viral replication. These results also showed that it does not affect the dysregulation of cellular gene splicing, ruling out its potential genotoxic effects [120]. A 50 mg dose of obefazimod effectively induced endoscopic remission, and histopathological scores improved after approximately 8 weeks of treatment. Further observations shown that long-term use of this drug at a dose of 50 mg daily effectively reduces symptoms associated with UC and maintains clinical remission. Additionally, determination of miR-124 expression in rectal biopsies and blood samples from patients participating in the study revealed a significant increase in miR-124 copy numbers compared to the placebo group. The most commonly reported adverse events included nausea and headache, although these were transient. A few cases of infections, tonsillitis, and oral herpes were reported [121]. In summary, obefazimod significantly reduces the intensity and extent of inflammatory changes, allowing patients with UC to achieve symptomatic, clinical, and endoscopic remission. The recently finished Phase 3 ABTECT programs (NCT05507203, NCT05507216) confirms the safety and efficacy of obefazimod, with a significant number of participants achieving clinical remission of UC, the results are waiting to be published, along with the completion of the long-term maintenance phase (NCT05535946) [126]. Additionally, obefazimod unique mechanism of action and convenient administration form speaks for itself. However, there is still a significant clinical need to revolutionize the approach to therapeutic strategies in IBD. Currently, the proposal for advanced combination therapy (ACT), which involves the simultaneous administration of at least two biological agents from different therapeutic groups, e.g., a monoclonal antibody and a small molecule drug, appears to be a step forward. Nevertheless, molecules that interact with miRNAs hold great promise, especially for patients who have not responded to current treatments, further emphasizing the need for continuous research into this type of therapeutic approach [121,125].

## 5. Final Conclusions

Advances in UC treatment are focusing on targeted therapies, extending beyond traditional steroids to novel biologic drugs, such as anti-TNFs, anti-integrins, anti-IL-12/23 agents, and small molecules, like JAK inhibitors and S1P modulators, which promote improved mucosal healing and reduced systemic side effects. Biosimilars are also being developed and are widely available, offering hope to those who fail to respond to traditional treatments and reducing the need for surgery. The potential of new molecules interacting with miRNAs holds great promise and future studies and clinical trials are needed to establish better and safer therapies.

## Figures and Tables

**Table 1:** Summary of conventional therapies in case of mild, moderate and severe ulcerative colitis.

Clasess of Drugs	Examples	Indications	Route of Administration	Drug Type/Release	Drug Elimination	Adverse Effects
5-aminosalicylic acid; 5-ASA	Sulfasalazine; SASP	mild to moderate UC	oral	produrg/cleavage by intestinal microbiome	kidneys (urine) and liver (bile)	nausea, vomiting, dyspepsia, appetite loss or headaches—mainly due to intolerance to the sulfapyridine component (carrier) [13]
	Mesalazine; Mesalamine	mild to moderate UC	oral and rectal	active moiety of sulphasalazine	kidneys (urine) and liver (bile)	headache, abdominal pain, nausea, diarrhoea, rash inflammatory reactions, pancreatitis, cardiotoxicity, hepatotoxicity, musculoskeletal complaints, respiratory symptoms, nephropathies and sexual dysfunction [16,23,24]
Glucocorticoids	Prednisone	moderate to severe UC	oral	prodrug/converted to prednisolone in the liver	kidneys (urine)	fatigue, headaches, mood changes, appetite changes/weight gain, swelling, increased risk of infection, hypertension, hyperglycemia, changes in lipid profile, disturbances in the metabolism of calcium and other electrolytes [26,27,29]
	Hydrocortisone	severe UC	intravenous	active drug	kidneys (urine)	fluid retention, weight gain, increased risk of infection, hypertension, hyperglycemia, changes in lipid profile, disturbances in the metabolism of calcium and other electrolytes [26,27,29]
	Methylprednisolone	severe UC	intravenous	active drug	kidneys (urine)	fluid retention, weight gain, increased risk of infection, hypertension, hyperglycemia, changes in lipid profile, disturbances in the metabolism of calcium and other electrolytes [26,27,29]
	Beclomethasone dipropionate; BDP	mild to moderate distal UC	oral and rectal	prodrug/activated in the intestine (and liver) by esterase enzymes	liver (bile) and kidneys (urine)	headache, nausea, dizziness, mood changes, skin darkening; without severe side effects [26,27,29]
	Budesonide	mild to moderate UC; left-sided UC	oral and rectal	involves prodrug strategies or targeted delivery systems (MMX)/pH-dependent system	liver (bile) and kidneys (urine)	headache, raised body temperature, insomnia, back pain, nausea, abdominal pain, diarrhoea, flatulence, nasopharyngitis [26,27,29,31]
Thiopurines	Azathioprine (AZA) and 6-mercaptopurine (6-MP)	steroid-refractory moderate to severe UC;	oral	Azathioprine is a prodrug of 6-mercaptopurine;	kidneys (urine) as secondary metabolites	leukopenia, bone marrow suppression, hepatotoxicity and kidney damage, gastric disorders, and pancreatitis. Long-term therapy has been associated with an increased risk of non-melanoma skin cancer, lymphoma, and cervical cancer [29,40]

UC—ulcerative colitis.

**Table 2:** Summary of antibodies used in the treatment of ulcerative colitis.

Generic Name	Type, Class and Subclass	Dosage Form	Mechanism of Action	Reference
Infliximab	chimeric human-mouse IgG1 monoclonal antibody	injection for intravenous infusion	antirheumatics, TNF alfa inhibitors	[29,42]
Adalimumab	fully human recombinant IgG1 antibody	subcutaneous injection	an immunosuppressive drug directed against TNF-α,	[29,47]
Vedolizumab	humanized monoclonal IgG1 antibody against integrin α4β7	intravenous infusion	specifically targets gut-tropic α4β7 anti-inflammatory activity	[29,52]
Etrolizumab	humanized monoclonal antibody against the β7 subunit of α4β7 and αEβ7 integrins	intravenous injection; subcutaneous injection	β7 subunit of α4β7 and αEβ7 integrin heterodimers	[53]
Golimumab	fully human monoclonal antibody of the IgG1қ class	subcutaneous injection	preventing TNF-α from binding to its receptors.	[61,62]
Ustekinumab	fully human monoclonal antibody of the IgG1қ class	administered intravenously during induction, subcutaneous formulation in maintenance	inhibiting activity of interleukin IL-12 and IL-23 leading to a reduction in inflammation.	[66,67]
Guselkumab	fully human monoclonal antibody of the IgG1λ class	administered intravenously during induction, subcutaneous formulation in maintenance	inhibiting activity of IL-23, a regulatory cytokine that influences the differentiation, expansion, and survival of T lymphocyte subsets	[70,71,72]
Mirikizumab	humanized monoclonal antibody of the IgG4 class	administered intravenously during induction, subcutaneous formulation in maintenance	inhibiting the interaction of human IL-23 cytokine and its receptor.	[72,73]

## Data Availability

No new data were created or analyzed in this study. Data sharing is not applicable to this article.

## Abbreviations

5-ASA	5-aminosalicylic acid
6-MMP	6-methylmercaptopurine
6-MP	6-mercaptopurine
6-TGN	6-thioguanine nucleotide
6-TUA	6-thiouric-acid
AM	adrenomodulin
ANCA	antineutrophil cytoplasmic antibodies
API	active pharmaceutical ingredients
ASCA	anti-Saccharomyces cerevisiae antibodies
ASUC	acute severe ulcerative colitis
ATP	adenosine triphosphate
AZA	Azathioprine
cAMP	cyclic adenosine monophosphate
CCBs	calcium channel blockers
CES1	carboxylesterase 1
CES2	carboxylesterase 2
CHMP	Committee for Medicinal Products for Human Use
CIR	controlled-release formulation
CRLR	calcitonin-like receptor
CRP	C-reactive protein
DAMP	damage-associated molecular pattern
DSS	dextran sodium sulphate
ECM	extracellular matrix
EMA	European Medicines Agency
ESR	erythrocyte sedimentation rate
FC	fecal calprotectin
FDA	The Food and Drug Administration
GLM	Golimumab
GM-CSF	granulocyte-macrophage colony-stimulating factor
GWAS	genome-wide association study
HIF-1	hypoxia-inducible factor 1
HLA	human leukocyte antigen
HNF4α	hepatocyte nuclear factor
IBD	inflammatory bowel disease
IC50	half-maximal inhibitory concentration
ICAM-1	intercellular adhesion molecule
IFX	Infliximab
IgG1	immunoglobulin G1
IKK	I-kB kinase
IL-23R	interleukin 23 receptor
ILC	innate lymphoid cells
iNOS	inducible nitric oxide synthase
IPAA	ileal pouch–anal anastomosis
JAK	janus activated kinases
JAK/STAT	janus kinase-signal transducer and activator of transcription protein
LFA-1	integrin 1 associated with leukocyte function
MAdCAM-1	mucosal vascular addressin cell adhesion molecule 1
MCP-1	monocyte chemoattractant protein-1
MES	Mayo Endoscopic Subscore
MMPs	matrix metalloproteinases
MMX	multi-matrix system
MUC2	mucin 2
NF-κB	nuclear factor kappa-light-chain-enhancer of activated B cells
Nfr2	Nuclear factor erythroid 2-related factor 2
NK	natural killers
NLRP3	nucleotide-binding domain, leucine-rich–containing family, pyrin domain–containing protein 3
NO	nitric oxide
NOAEL	no-observed-adverse-effect level
NOTCH	neurogenic locus notch homolog protein
PAMP	pathogen-associated molecular pattern
pANCA	perinuclear anti-neutrophil cytoplasmic antibodies
PCR	polymerase chain reaction
PDE4	phosphodiesterase-4
PGE2	prostaglandin E2
PGI2	prostaglandin I2
PI3K/Akt	phosphatidylinositol 3-kinase-protein kinase
PML	progressive multifocal leukoencephalopathy
PPAR	peroxisome proliferator-activated receptor
PSC	primary sclerosing cholangitis
RAMP	receptor activity-modifying protein
S1P	sphingosine-1-phosphate
SASP	Sulfasalazine
SCFA	short-chain fatty acids
SP1R1	sphingosine-1-phosphate receptor
SPL	S1P lyase
STAT3	signal transducer and activator of transcription 3
TACE	TNF-α converting enzyme
TIMPs	tissue inhibitors of metalloproteinases
TLR	toll-like receptor
TMS	transcranial magnetic stimulation
TNBS	2,4,6-Trinitrobenzene sulfonic acid
TNF-α	tumor necrosis factor alpha
TPMT	thiopurine S-methyltransferase
TK	tyrosine kinase
UC	ulcerative colitis
UCEIS	Ulcerative Colitis Endoscopic Index of Severity
UPA	upadacitinib
VCAM-1	vascular cell adhesion molecule 1
VEGF	vascular endothelial growth factor
XO	xanthine oxidase
